# Supplementary material for: Observation of optical gyromagnetic properties in a magneto-plasmonic metamaterial
Source: Nat Commun. 2022 Mar 31;13:1719. doi: 10.1038/s41467-022-29452-9 (PMC8971533; doi:10.1038/s41467-022-29452-9)
Supplement: Supplementary file 1 — Supplementary Information [file 41467_2022_29452_MOESM1_ESM.pdf]

**Supplementary Information**

# Observation of optical gyromagnetic properties in a magneto-plasmonic metamaterial

Weihao Yang,[1§] Qing Liu,[2§] Hanbin Wang,[3§] Yiqin Chen,[2§] Run Yang,[1] Shuang Xia,[1] Yi Luo,[3] Longjiang Deng,[1] Jun Qin,[1*] Huigao Duan,[2*] and Lei Bi[1*]

[1]National Engineering Center of Electromagnetic Radiation Control Materials, School of Electronic Science and Engineering, University of Electronic Science and Technology of China, Chengdu, China, 610054

[2]College of Mechanical and Vehicle Engineering, Hunan University, Changsha 410082, China

[3]Microsystem and Terahertz Research Center, China Academy of Engineering Physics, Chengdu 610200, China

E-mail: qinjun@uestc.edu.cn, duanhg@hnu.edu.cn, bilei@uestc.edu.cn

§ These authors contributed equally

**Supplementary Note 1: X-ray diffraction (XRD) and Faraday rotation of the Ce:YIG/YIG films on silicon**

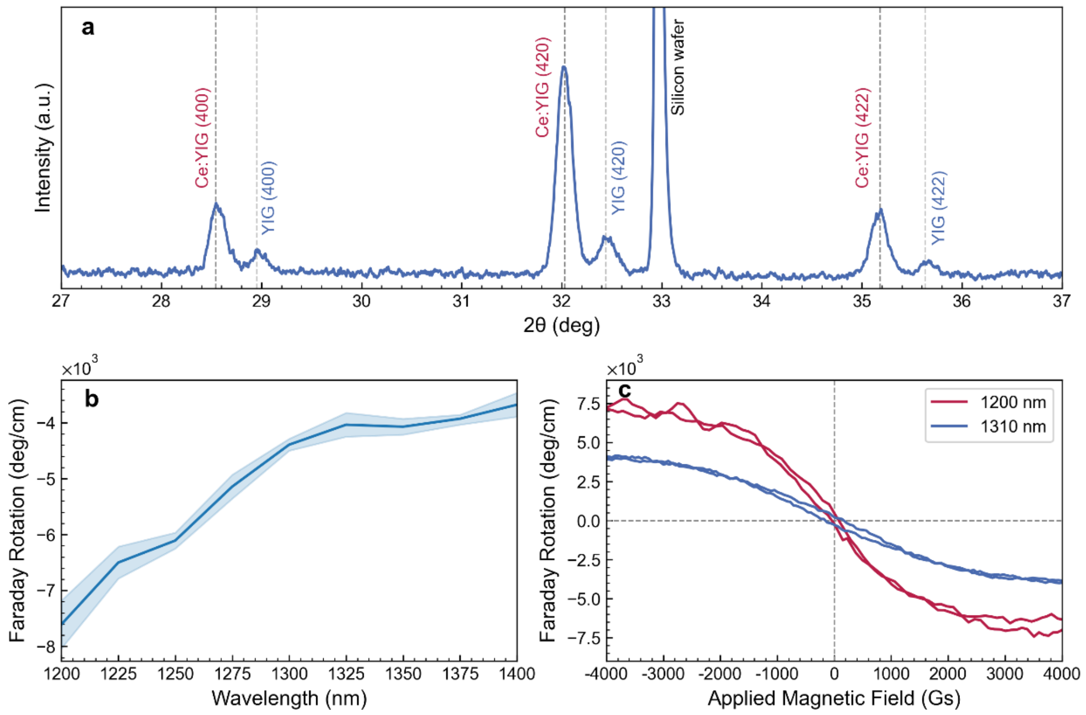


**Supplementary Figure 1. XRD and Faraday rotation under normal incidence. a**, Measured XRD spectra of the Ce:YIG/YIG films on a Si substrate. **b**, Measured Faraday rotation spectra of the Ce:YIG/YIG films on Si under normal incidence. **c**, Measured Faraday rotation hysteresis of the Ce:YIG/YIG films on Si at 1200 nm and 1310 nm wavelengths.

**Supplementary Note 2: Simulated and experiment reflection spectra under different incident angles in the orthogonal (XZ) plane.**

Supplementary Figure 2a and 2b show the reflectance spectra for p-polarized incidence in XZ plane with incident angles ranging from 45° to 70° of experiment and simulation. For p-polarization, part of the electric field is along the top horizontal arm direction, therefore both the electric quadrupole and magnetic dipole resonances can be excited. As increasing the incident angle from 45° to 70°, both the reflectivity at the electric quadrupole and magnetic dipole resonances are decreased, due to the decrease of the *x*-component electric fields. Both resonances show almost no wavelength shift, because only the *x*-component electric field contributes to the excitation of the two resonances. The experiment results are consistent with our simulation. However, the experimental magnetic resonance shows a little blue-shifts as increasing the incident angles. This may be resulted from the asymmetric gap area of the fabricated SRR structures, as shown in the inset of Fig. 1c of the manuscript. For s-polarization shown in

Supplementary Figure 2c and 2d, the electric field is along the two vertical arm direction. For this situation, both the symmetric and antisymmetric electric resonance modes are excited at ~900 nm and ~1000 nm wavelengths respectively. For oblique incidence, the modes excited at two vertical arms will experience a small time-delay. The near field in the two vertical arms are different, leading to the occurrence of the antisymmetric mode. Therefore, as increasing the incident angles, one electric resonance will split into a red-shifted symmetric mode and a blue-shifted antisymmetric mode, as shown in Supplementary Figure 2c. The displacement current directions of the symmetric mode are shown in the inset of the Supplementary Figure 2c. In Supplementary Figure 2d, we simulated the reflectance spectra under s-polarization, which are consistent with our experiment results. The displacement current directions of the asymmetric mode are shown in the inset of the Supplementary Figure 2c.

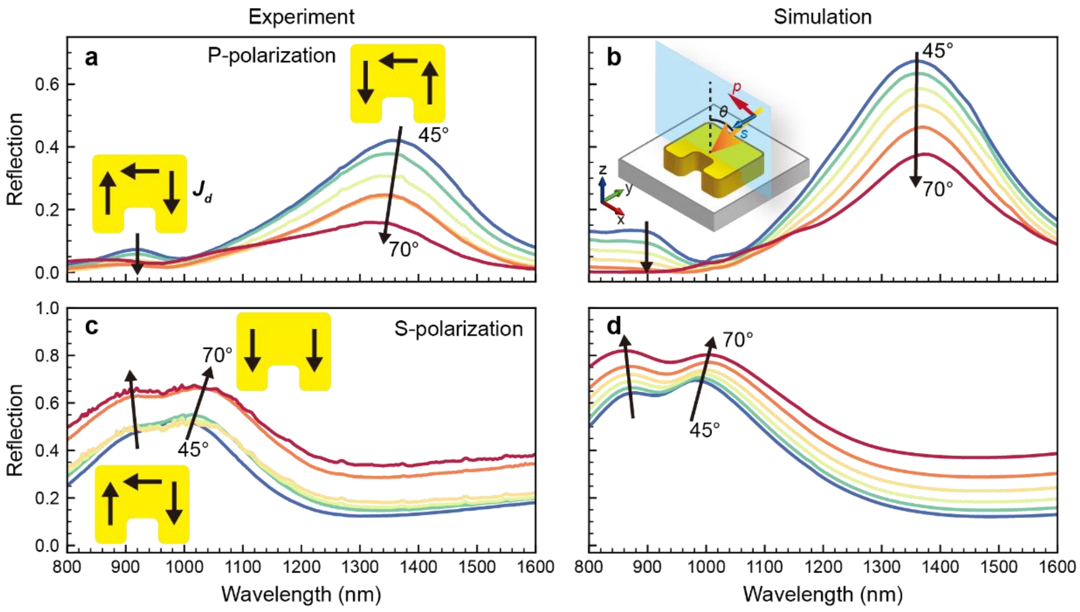


**Supplementary Figure 2. Reflection spectra under p and s-polarized incidence in the XZ plane a**, Measured and **b**, Simulated reflection spectra for incident angles ranging from 45° to 70° under p-polarized incidence. The inset of a shows the displacement current directions in the SRR. The inset of b shows the schematic of the incident plane and polarization. **c**, Measured and **d**, simulated reflection spectra for incident angles ranging from 45° to 70° under s-polarized incidence. The inset of **c** shows the displacement current directions in the SRR.

## Supplementary Note 3: Transmission spectra under normal incidence

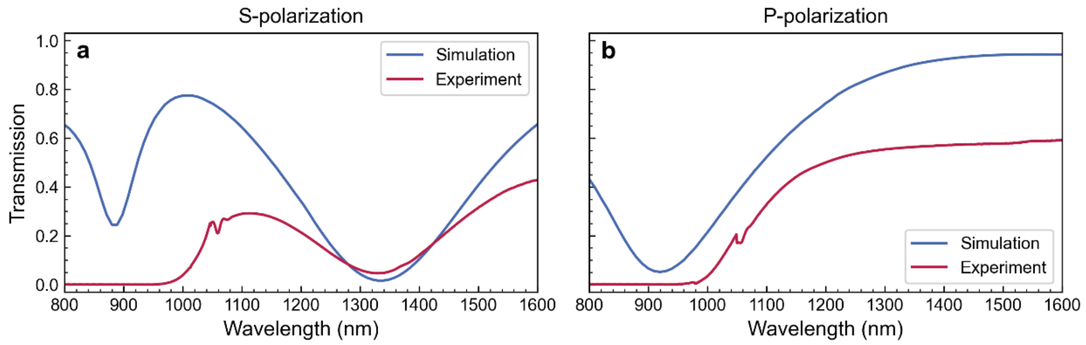


**Supplementary Figure 3. Transmission spectra under normal incidence. a**, Measured and simulated transmission spectra of the MO-SRR metamaterial for normal s-polarized incidence (electric field parallel to the gap of SRR). **b**, Transmission spectra of the MO-SRR metamaterial for p-polarized incidence (electric field perpendicular to the gap of SRR). Compared to simulation, the experimentally observed broader resonance peak at 1340 nm wavelength for s-polarized incidence may be attributed to the imperfect sample geometry due to the nanofabrication process. The low transmission in experiments for 1100 nm and shorter wavelengths is due to the absorption of the thick silicon substrate.

## Supplementary Note 4: The step-by-step derivation of relationship between TMOKE and gyroelectric and gyromagnetic tensors.

In order to clarify the TMOKE in metamaterials with bi-gyrotropic properties, we provide a detailed theoretical analysis starting from the Maxwell's equations. Firstly, let's consider a bi-gyrotropic medium with external magnetic field along the x direction, as shown in Supplementary Figure 4a.

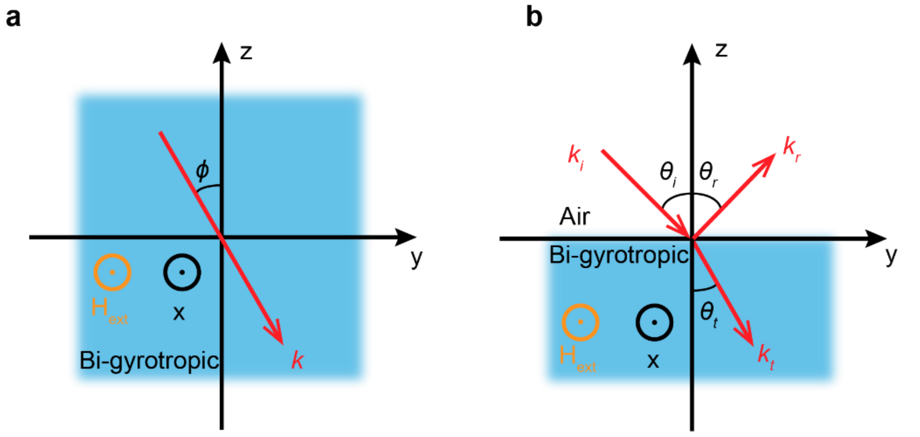


**Supplementary Figure 4. Electromagnetic wave propagation in a bi-gyrotropic material. a**, Schematic diagram of electromagnetic wave propagation in a bi-gyrotropic material. **b**, Schematic diagram of the reflection and refraction of electromagnetic waves at the interface between a bi-gyrotropic material and air. The applied magnetic field is along the x-axis direction.

The permittivity and permeability tensors of such a medium take the form[1-3]

$$\overset{=}{\varepsilon} = \begin{bmatrix} \varepsilon_x & 0 & 0 \\ 0 & \varepsilon & -j\gamma \\ 0 & j\gamma & \varepsilon \end{bmatrix}, \overset{=}{\mu} = \begin{bmatrix} \mu_x & 0 & 0 \\ 0 & \mu & -j\kappa \\ 0 & j\kappa & \mu \end{bmatrix} \qquad (1)$$

where $\varepsilon$ and $\mu$ are the diagonal components of the permittivity and permeability, $\gamma$ and $\kappa$ are the off-diagonal components of permittivity and permeability. The electric and magnetic field vectors can be expressed as $\vec{E} = E_0 e^{-j\vec{k}\cdot\vec{r}}, \vec{H} = H_0 e^{-j\vec{k}\cdot\vec{r}}$. Therefore, the Maxwell's equation $\nabla \cdot \vec{B} = 0, \nabla \cdot \vec{D} = 0$ can be written as

$$\begin{aligned} \vec{k} \times \vec{E} - k_0 \overset{=}{\mu} \vec{H} = 0 \\ \vec{k} \times \vec{H} + k_0 \overset{=}{\varepsilon} \vec{E} = 0 \end{aligned} \qquad (2)$$

Where $k_0 = \omega / c$ is the free space wave vector, $\vec{k} = [k_x, k_y, k_z]^T$ is the wave vector in the bi-gyrotropic medium. In the following derivations, we consider the incident plane perpendicular to the external magnetic field, *i.e.* the Voigt geometry. This is also the geometric relationship between the applied magnetic field and the wave vector for the TMOKE.

Consider the case of a homogeneous, bi-gyrotropic medium in Supplementary Figure 4a, we have $\vec{k} = [0, k\sin\phi, -k\cos\phi]^T$, $\vec{E} = [0, E_y, E_z]^T$, $\vec{H} = [H_x, 0, 0]^T$ for p-polarization and $\vec{k} = [0, k\sin\phi, -k\cos\phi]^T, \vec{E} = [E_x, 0, 0]^T, \vec{H} = [0, H_y, H_z]^T$ for s-polarization. Here, $\phi$ is the incident angle with respect to the $z$ axis in the medium, **k** is the wavevector in the bi-gyrotropic medium, and $n_s$, $n_p$ is the refractive index of the medium under s and p-polarization. Then equation (2) can be factorized into two equations for s and p polarizations:

$$\begin{aligned} &\varepsilon_x E_x + \frac{k}{k_0}\cos\phi \cdot H_y + \frac{k}{k_0}\sin\phi \cdot H_z = 0 \\ &\frac{k}{k_0}\cos\phi E_x + \mu H_y - j\kappa H_z = 0 \quad \text{(s-polarization)} \\ &\frac{k}{k_0}\sin\phi E_x + j\kappa H_y + \mu H_z = 0 \end{aligned} \qquad (3)$$

$$\mu_x H_x - \frac{k}{k_0}\cos\phi \cdot E_y - \frac{k}{k_0}\sin\phi \cdot E_z = 0$$

$$-\frac{k}{k_0}\cos\phi H_x + \varepsilon E_y - j\gamma E_z = 0 \quad \text{(p-polarization)} \qquad (4)$$

$$-\frac{k}{k_0}\sin\phi H_x + j\gamma E_y + \varepsilon E_z = 0$$

From equations (3), we see only the $\mu$, $\kappa$ and $\varepsilon$ components are present, while $\gamma$ is absent from the equations for s-polarization. Whereas only the $\mu$, $\varepsilon$ and $\gamma$ components are present, while $\kappa$ is absent from the equations for p-polarization. Given the homogeneous equation (3) and (4), they will have non-trivial solutions when the determinant of the coefficients vanishes:

$$\begin{vmatrix} \varepsilon_x & \frac{k}{k_0}\cos\phi & \frac{k}{k_0}\sin\phi \\ \frac{k}{k_0}\cos\phi & \mu & -j\kappa \\ \frac{k}{k_0}\sin\phi & j\kappa & \mu \end{vmatrix} = 0 \quad \text{(s-polarization)} \qquad (5)$$

$$\begin{vmatrix} \mu_x & -\frac{k}{k_0}\cos\phi & -\frac{k}{k_0}\sin\phi \\ -\frac{k}{k_0}\cos\phi & \varepsilon & -j\gamma \\ -\frac{k}{k_0}\sin\phi & j\gamma & \varepsilon \end{vmatrix} = 0 \quad \text{(p-polarization)} \qquad (6)$$

Thus, we have obtained the generalized formulas for the Cotton-Mouton effect in a bi-gyrotropic medium[4]:

$$\begin{aligned} n_s^2 &= \varepsilon_x \mu (1 - Q_m^2) \\ n_p^2 &= \varepsilon \mu_x (1 - Q^2) \end{aligned} \qquad (7)$$

Where $Q=\gamma/\varepsilon$, $Q_m=\kappa/\mu$. In addition, for s-polarization, the magnetic field of the wave can be found:

$$\vec{H} = H_0 \begin{bmatrix} 0 \\ \cos\phi + jQ_m \sin\phi \\ \sin\phi - jQ_m \cos\phi \end{bmatrix} \qquad (8)$$

where $H_0$ is magnetic field amplitude. This difference in refractive index for different polarizations provides the possibility to distinguish the gyroelectric and gyromagnetic contributions of bi-gyrotropic materials.

Next, let us consider transmission and reflection of a light wave at the interface

between air and a bi-gyrotropic medium. As shown in Supplementary Figure 4b, a plane wave is incident from air ($n_1$=1) onto a bi-gyrotropic medium at the incident angle of $\theta_i$, with the electrical vectors perpendicular to the plane of incidence (s wave). The magnetic field of the incident and reflected waves are $\vec{H}_i = H_i[0, \cos\theta_i, \sin\theta_i]^T$, $\vec{H}_r = H_r[0, \cos\theta_r, -\sin\theta_r]^T$ , $\theta_r = \theta_i = \theta$ . According to equation (7) $n_2 = n_s = \sqrt{\varepsilon_x \mu(1 - Q_m^2)}$ and Snell's law $\sin\theta_t = (1/n_s)\sin\theta$, the magnetic field of the transmitted wave can be found from wave equation (8):

$$\vec{H}_t = H_t \begin{bmatrix} 0 \\ \cos\theta_t + jQ_m \sin\theta_t \\ \sin\theta_t - jQ_m \cos\theta_t \end{bmatrix} \quad (9)$$

The electric field of the transmitted wave take from $\vec{E} = \frac{1}{k_0} \vec{H} \times \vec{k}$. Considering the continuity of the tangential components of **E** and **H** at the interface, for s-polarization we have:

$$E_{ix} + E_{rx} = E_{tx}, H_{iy} + H_{ry} = H_{ty}$$

$$\begin{aligned} H_i - H_r &= H_t / n_s \\ (H_i + H_r)\cos\theta &= H_t(\cos\theta_t + jQ_m \sin\theta_t) \end{aligned} \quad (10)$$

Hence the formulae for the reflection coefficients in the linear approximation immediately follow:

$$\begin{aligned} r_s &= \frac{E_r}{E_i} = \frac{H_r}{H_i} = \frac{\cos\theta/n_s - \cos\theta_t + jQ_m \sin\theta_t}{\cos\theta/n_s + \cos\theta_t + jQ_m \sin\theta_t} \\ &\approx \frac{\cos\theta - n_s\cos\theta_t}{\cos\theta + n_s\cos\theta_t} + 2jQ_m \frac{\sin\theta\cos\theta}{(\cos\theta + n_s\cos\theta_t)^2} \end{aligned} \quad (11)$$

Similarly, the continuity of the tangential components of **E** and **H** at the interface, for p-polarization we have:

$$E_{iy}+E_{ry}=E_{ty}, H_{ix}+H_{rx}=H_{tx}$$
$$E_i-E_r=n_p E_t \qquad (12)$$
$$(E_i+E_r)\cos\theta=E_t(\cos\theta_t+jQ\sin\theta_t)$$

And the reflection coefficient under p-polarization should be expressed as

$$\begin{aligned} r_p=\frac{E_r}{E_i}&=\frac{n_p\cos\theta-\cos\theta_t+jQ\sin\theta_t}{n_p\cos\theta+\cos\theta_t+jQ\sin\theta_t} \\ &\approx\frac{n_p\cos\theta-\cos\theta_t}{n_p\cos\theta+\cos\theta_t}+2jQ\frac{\sin\theta\cos\theta}{(n_p\cos\theta+\cos\theta_t)^2} \end{aligned} \qquad (13)$$

Notice that when $Q$ or $Q_m$ is equal to 0 (a non-magnetic material), equation (11) and equation (13) agree with the Fresnel equations.

Abbreviating equation (11) and equation (13) as

$$\begin{aligned} r_s&=\tilde{r}_s(1+j\rho_s) \\ r_p&=\tilde{r}_p(1+j\rho_p) \end{aligned} \qquad (14)$$

Where $\tilde{r}_s=\frac{\cos\theta-n_s\cos\theta_t}{\cos\theta+n_s\cos\theta_t}$, $\rho_s=2Q_m\frac{\sin\theta\cos\theta}{\cos^2\theta-n_s^2\cos^2\theta_t}$, $\tilde{r}_p=\frac{n_p\cos\theta-\cos\theta_t}{n_p\cos\theta+\cos\theta_t}$,

$\rho_p=2Q\frac{\sin\theta\cos\theta}{n_p^2\cos^2\theta-\cos^2\theta_t}$.

One can determine the reflectivity ($R=|r|^2$) under positive and negative applied magnetic fields:

$$\begin{aligned} R_s(+H)&=|\tilde{r}_s(1+j\rho_s)|^2=|\tilde{r}_s[(1-\mathrm{Im}\rho_s)+j\mathrm{Re}\rho_s]|^2 \\ &=|\tilde{r}_s|^2[(1-\mathrm{Im}\rho_s)^2+(\mathrm{Re}\rho_s)^2] \\ R_s(-H)&=|\tilde{r}_s(1-j\rho_s)|^2=|\tilde{r}_s[(1+\mathrm{Im}\rho_s)-j\mathrm{Re}\rho_s]|^2 \\ &=|\tilde{r}_s|^2[(1+\mathrm{Im}\rho_s)^2+(\mathrm{Re}\rho_s)^2] \\ R_p(+H)&=|\tilde{r}_p(1+j\rho_p)|^2=|\tilde{r}_p[(1-\mathrm{Im}\rho_p)+j\mathrm{Re}\rho_p]|^2 \\ &=|\tilde{r}_p|^2[(1-\mathrm{Im}\rho_p)^2+(\mathrm{Re}\rho_p)^2] \\ R_p(-H)&=|\tilde{r}_p(1-j\rho_p)|^2=|\tilde{r}_p[(1+\mathrm{Im}\rho_p)-j\mathrm{Re}\rho_p]|^2 \\ &=|\tilde{r}_p|^2[(1+\mathrm{Im}\rho_p)^2+(\mathrm{Re}\rho_p)^2] \end{aligned} \qquad (15)$$

Finally, we obtain the TMOKE:

$$\delta_s = 2\frac{R_s(+H) - R_s(-H)}{R_s(+H) + R_s(-H)} = \frac{-8|\tilde{r}_s|^2 \mathrm{Im}\rho_s}{2|\tilde{r}_s|^2(1+|\rho_s|^2)} = -4\mathrm{Im}\frac{\rho_s}{1+|\rho_s|^2}$$

$$\delta_p = 2\frac{R_p(+H) - R_p(-H)}{R_p(+H) + R_p(-H)} = \frac{-8|\tilde{r}_p|^2 \mathrm{Im}\rho_p}{2|\tilde{r}_p|^2(1+|\rho_p|^2)} = -4\mathrm{Im}\frac{\rho_p}{1+|\rho_p|^2} \quad (16)$$

Ultimately, we can conclude that the TMOKE of s-polarization is only related to the off-diagonal elements of the permeability tensor, and the TMOKE of p-polarization is only related to the off-diagonal elements of the permittivity tensor.

**Supplementary Note 5: Simulated s-polarized TMOKE of the metamaterial under different incident angles**

As increasing the incident angles, the TMOKE of the structure under s-polarization increases first and then decreases, with a maximum value of $2.7\times10^{-3}$ at 30°. Due to the angle limitation of the ellipsometer (45°-75°), the strongest s-polarized TMOKE was experimentally measured at 45° incidence.

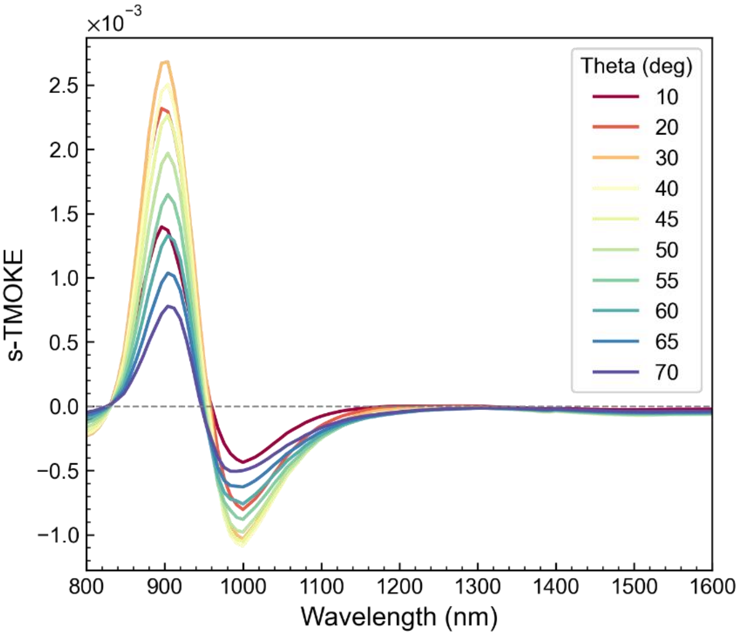


**Supplementary Figure 5. Simulated s-polarized TMOKE of the metamaterial.** The simulation result shows clear TMOKE signal up to $10^{-3}$ level of the MO-SRR metamaterial, for different incident angles ranging from 10° to 70° under s-polarized incidence.

**Supplementary Note 6: P-polarized TMOKE of the Ce:YIG/YIG films on silicon**

We measured the reflection and TMOKE spectra of the bare Ce:YIG/YIG films on Si, as shown in Supplementary Figure 6b. The TMOKE exists only for p-polarized

incidence. Although the reflectivity for s-polarization is 0.2 under 45° incidence, which is even smaller than the metamaterial, its s-polarized TMOKE is still 0 due to a unity μ tensor of the MO materials. We notice a large p-polarized TMOKE of 0.17 at 850 nm wavelength. This is due to a low reflectivity ~0 of the thin films at around the Brewster angle, causing an optical enhancement of the TMOKE.

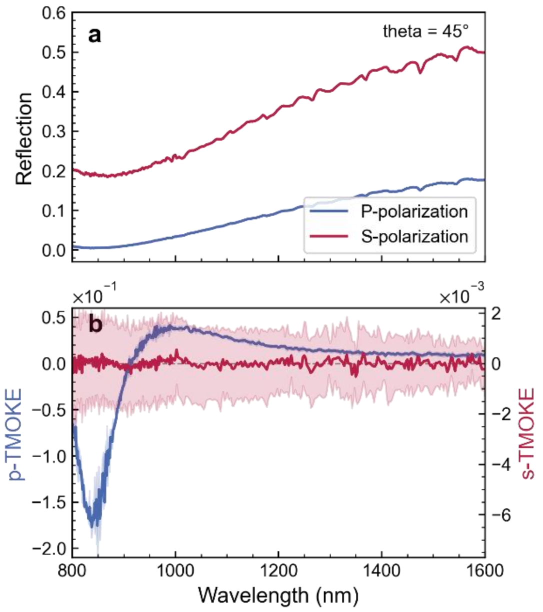


**Supplementary Figure 6. Reflection and TMOKE spectra under 45° incidence of the bare film. a**, reflection spectra of the Ce:YIG/YIG films on Si under s- and p-polarized incidence. **b**, TMOKE spectra of the Ce:YIG/YIG films on Si under s- and p-polarized incidence.

## Supplementary Note 7: The step-by-step derivation of metamaterial equivalent material parameters retrieval method

The incident plane is *x*-*z* plane, the electric (magnetic) field vector is parallel to the *y* axis for s- (p-) polarization. The equivalent material structure is shown in Supplementary Figure 7b. The thickness of the equivalent material is set to the thickness of the part that does not contain the substrate and air (the distance from the lower surface of the YIG to the upper surface of the gold is 155 nm), and the upper and lower parts of the equivalent material are set to semi-infinite space of air and substrate, respectively. Firstly, we use COMSOL software to simulate the transmission and reflection coefficient of the MO SRR metamaterial. In our COMSOL model, the energy port is needed to set at a certain distance away from the device to avoid the influence of the port on the field distributions, this distance is usually taken to be greater than the maximum wavelength. Therefore, in our simulation, the distance between incident port and the upper surface of gold is $d_1$, and the distance between outgoing port and the

lower surface of YIG is $d_2$, in our model $d_1 = d_2 = 2$ μm, as shown in Supplementary Figure 7b. Since the complex S matrix contains phase information (distance-dependent), the phase change caused by the port needs to be corrected by the following equation[5]:

$$\begin{bmatrix} S_{11} & S_{21} \\ S_{12} & S_{22} \end{bmatrix} = \begin{bmatrix} e^{-jk_a d_1} & 0 \\ 0 & e^{-jk_s d_2} \end{bmatrix} \begin{bmatrix} S'_{11} & S'_{21} \\ S'_{12} & S'_{22} \end{bmatrix} \begin{bmatrix} e^{-jk_a d_1} & 0 \\ 0 & e^{-jk_s d_2} \end{bmatrix} \tag{17}$$

[$S$'] is the raw scattering matrix between the two ports, and $k_a = \frac{2\pi}{\lambda}\cos\theta$, $k_s = \sqrt{\left(\frac{2\pi}{\lambda_s}\right)^2 - k_t^2}$ are the wave numbers along the z-axis direction in air and substrate, where $k_t = \frac{2\pi}{\lambda}\sin\theta$, $\lambda$ and $\lambda_s$ are the wavelengths in air and substrate. The modified parameters are only relevant to the metamaterial itself, and the aforementioned values of $d_1$, $d_2$ can be taken as any value greater than the maximum wavelength. The corrected $S_{21}$ and $S_{11}$ are corresponding to the transmission ($T$) and reflection ($R$). Then, based on the 2×2 transfer matrix method[6-8], the transmission and reflection coefficients of the structure shown in Supplementary Figure 7b can be obtained as a function of its material parameters:

$$T = \frac{2\alpha_a k_a A}{(\alpha_a k_a + \alpha_s k_s)\cos(kd) - i\left(\alpha_m k + \frac{\alpha_a k_a \alpha_s k_s}{\alpha_m k}\right)\sin(kd)} \tag{18}$$

$$R = \frac{(\alpha_a k_a - \alpha_s k_s)\cos(kd) + i\left(\alpha_m k - \frac{\alpha_a k_a \alpha_s k_s}{\alpha_m k}\right)\sin(kd)}{(\alpha_a k_a + \alpha_s k_s)\cos(kd) - i\left(\alpha_m k + \frac{\alpha_a k_a \alpha_s k_s}{\alpha_m k}\right)\sin(kd)} \tag{19}$$

Where $k_a$, $k$, $k_s$ are the wave numbers along the z-axis direction in air, metamaterial and substrate. The coefficients $\alpha_{a,m,s}$ and A are dependent on the polarization and defined as:

$$\begin{aligned} &\mathrm{S-polarization:}\ \alpha_{a,m,s} = \frac{1}{\mu_{a,m,s}},\quad A = 1 \\ &P-\mathrm{polarization:}\ \alpha_{a,m,s} = \frac{1}{\varepsilon_{a,m,s}}, A = \frac{1}{n_s} \end{aligned} \tag{20}$$

*a, m, s* stands for air, metamaterial, substrate respectively, $n_s$ is the index of substrate. Define abbreviations $k_{a,s} = \alpha_{a,s}k_{a,s}$, $\xi = \alpha_m k$. After using these abbreviations, $T/R$ can be written as:

$$T = \frac{2k_a \xi A}{\xi(k_a + k_s)\cos(kd) - i(\xi^2 + k_a k_s)\sin(kd)} \tag{21}$$

$$R=\frac{\xi(k_a-k_s)\cos(kd)+i(\xi^2-k_ak_s)\sin(kd)}{\xi(k_a+k_s)\cos(kd)-i(\xi^2+k_ak_s)\sin(kd)} \tag{22}$$

By inverse operation, the wave number and impedance coefficients $\xi$ in metamaterials can then be obtained by *T/R* from the simulated results:

$$kd=\pm\,\mathrm{arccos}\left(\frac{k_a(1-R^2)+k_s(T/A)^2}{(T/A)\left[k_a(1-R)+k_s(1+R)\right]}\right)+2m\pi \tag{23}$$

$$\xi=\pm\sqrt{\frac{k_a^2(R-1)^2-k_s(T/A)^2}{(R+1)^2-(T/A)^2}} \tag{24}$$

The sign is determined by the specific physical law, $+2m\pi$ is to make the value of the equation continuous. Since the wave vector is related to the material parameters, the equivalent material parameters can be derived from the following equation:

$$\begin{aligned}&\mathrm{S-polarization}\colon\ \mu=\frac{k}{\xi},\varepsilon=\frac{(k_t^2+k^2)c^2\xi}{(2\pi f)^2k}\\&\mathrm{P-polarization}\colon\ \varepsilon=\frac{k}{\xi},\mu=\frac{(k_t^2+k^2)c^2\xi}{(2\pi f)^2k}\end{aligned} \tag{25}$$

In particular, the equivalent material parameters of the metamaterials appear to have a negative imaginary part, which does not imply energy amplification, but rather indicates the transfer of energy between the magnetic and electric fields[9-12]. $\mathrm{Im}(\varepsilon)<0$ means that energy transfer from the magnetic to the electric field, and $\mathrm{Im}(\mu)<0$ means that energy transfer from the electric to the magnetic field.

After achieving the diagonal elements of the $\varepsilon$ and $\mu$, we use the 4×4 transfer matrix method to retrieve the off-diagonal elements. In our method, the equivalent layer is expressed by the fully anisotropic materials. Then, the Maxwell's equations can be written as the matrix differential equation[9]:

$$\frac{\partial}{\partial z'}\begin{bmatrix}E_x\\E_y\\\tilde{H}_x\\\tilde{H}_y\end{bmatrix}=\begin{bmatrix}-j(\tilde{k}_y\frac{\mu_{yz}}{\mu_{zz}}+\tilde{k}_x\frac{\varepsilon_{zx}}{\varepsilon_{zz}}) & j\tilde{k}_x(\frac{\mu_{yz}}{\mu_{zz}}-\frac{\varepsilon_{zy}}{\varepsilon_{zz}}) & (\frac{\tilde{k}_x\tilde{k}_y}{\varepsilon_{zz}}+\mu_{yx}-\frac{\mu_{yz}\mu_{zx}}{\mu_{zz}}) & (-\frac{\hat{k}_x^2}{\varepsilon_{zz}}+\mu_{yy}-\frac{\mu_{yz}\mu_{zy}}{\mu_{zz}})\\ j\tilde{k}_y(\frac{\mu_{xz}}{\mu_{zz}}-\frac{\varepsilon_{zx}}{\varepsilon_{zz}}) & -j(\tilde{k}_x\frac{\mu_{xz}}{\mu_{zz}}+\tilde{k}_y\frac{\varepsilon_{zy}}{\varepsilon_{zz}}) & (\frac{\hat{k}_y^2}{\varepsilon_{zz}}-\mu_{xx}+\frac{\mu_{xz}\mu_{zx}}{\mu_{zz}}) & (-\frac{\tilde{k}_x\tilde{k}_y}{\varepsilon_{zz}}-\mu_{xy}+\frac{\mu_{xz}\mu_{zy}}{\mu_{zz}})\\ (\frac{\tilde{k}_x\tilde{k}_y}{\mu_{zz}}+\varepsilon_{yx}-\frac{\varepsilon_{yz}\varepsilon_{zx}}{\varepsilon_{zz}}) & (-\frac{\hat{k}_x^2}{\mu_{zz}}+\varepsilon_{yy}-\frac{\varepsilon_{yz}\varepsilon_{zy}}{\varepsilon_{zz}}) & -j(\tilde{k}_y\frac{\varepsilon_{yz}}{\varepsilon_{zz}}+\tilde{k}_x\frac{\mu_{zx}}{\mu_{zz}}) & j\tilde{k}_x(\frac{\varepsilon_{yz}}{\varepsilon_{zz}}-\frac{\mu_{zy}}{\mu_{zz}})\\ (\frac{\hat{k}_y^2}{\mu_{zz}}-\varepsilon_{xx}+\frac{\varepsilon_{xz}\varepsilon_{zx}}{\varepsilon_{zz}}) & (-\frac{\tilde{k}_x\tilde{k}_y}{\mu_{zz}}-\varepsilon_{xy}+\frac{\varepsilon_{xz}\varepsilon_{zy}}{\varepsilon_{zz}}) & j\tilde{k}_y(\frac{\varepsilon_{xz}}{\varepsilon_{zz}}-\frac{\mu_{zx}}{\mu_{zz}}) & -j(\tilde{k}_x\frac{\varepsilon_{xz}}{\varepsilon_{zz}}+\tilde{k}_y\frac{\mu_{zy}}{\mu_{zz}})\end{bmatrix}\begin{bmatrix}E_x\\E_y\\\tilde{H}_x\\\tilde{H}_y\end{bmatrix} \tag{26}$$

Here, $z'=k_0z$, $\tilde{k}_{x/y/z}=\frac{k_{x/y/z}}{k_0}$, $\vec{\tilde{H}}=j\eta_0\vec{H}$, $k_0$ is the wave vector of free space, *z* is the direction of electromagnetic wave propagation, $\vec{E}$ and $\vec{H}$ are the electric and

magnetic field. Through solving the matrix differential equation, we can obtain the solution as:

$$\psi(z') = \mathrm{W}e^{\lambda z'}c \qquad (27)$$

$$\psi = \begin{bmatrix} E_x \\ E_y \\ \tilde{H}_x \\ \tilde{H}_y \end{bmatrix} \qquad (28)$$

Here, the W and $\lambda$ are the eigen-vector matrix and eigen-value matrix of the right-side 4×4 matrix in equation (26), $c = \mathrm{W}^{-1}\psi(0)$.

For multilayers, combined with the boundary conditions at both interfaces of each layer. We can achieve the transfer matrix:

$$\mathrm{T}_i = \mathrm{W}_{i+1}^{-1}\mathrm{W}_i e^{\lambda_i k_0 L_i} \qquad (29)$$

Where, $L_i$ is the thickness of the $i$ layer, $i$ is the positive integer.

According to above equations, we use the 4×4 transfer matrix to calculate the complex reflection coefficients of the multilayers shown in Supplementary Figure 7c. Here, the complex reflection coefficients are the functions of off-diagonal elements of $\varepsilon$ or $\mu$ tensors (s-polarization: $\mu$, p-polarization: $\varepsilon$). In order to deduce the off-diagonal elements, we first simulate the complex reflection coefficients of the MO-SRR metamaterial under positive and negative applied magnetic field using COMOSL. Then, we calculate the TMOKE spectrum according to the equation (2) in the manuscript, and phase difference defined as:

$$\Delta\text{phase} = a\tan\left(\frac{imag(R(H+))}{real(R(H+))}\right) - a\tan\left(\frac{imag(R(H-))}{real(R(H-))}\right) \qquad (30)$$

Here, $R(\mathrm{H}\pm)$ is the complex reflection coefficient under positive and negative magnetic field respectively. Finally, we use the least square method to fit the simulated TMOKE and phase difference by the 4×4 transfer matrix.

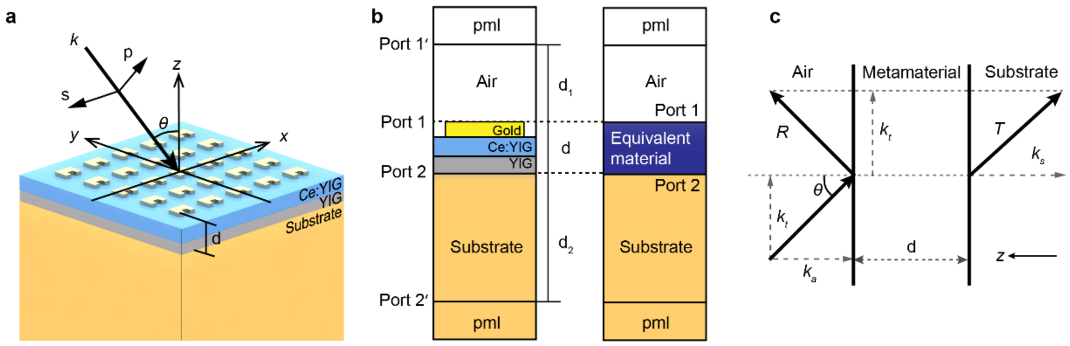

**Supplementary Figure 7. Schematic diagram of metamaterial structure and its equivalent material. a**, Illustration of the incident plane, angle and polarization relative to the metamaterial. **b**, Schematic diagram of the equivalent material in our simulation. **c**, Schematic diagram of the theoretical model for the derivation of the effective electromagnetic parameters.

## Supplementary Note 8: Transmission, reflection and TMOKE spectra of equivalent materials and MO-SRR metamaterial

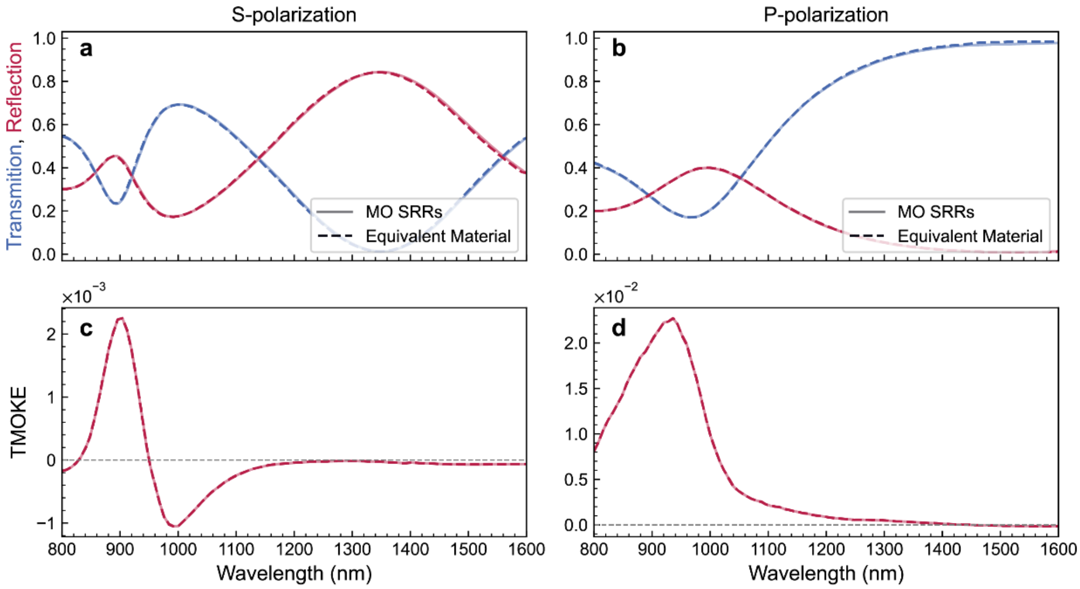


**Supplementary Figure 8. Transmission, reflection and TMOKE spectra under 45° incidence of equivalent materials. a** and **b**, Transmission and reflection spectra of MO SRR and equivalent material under s- and p-polarized incidence. **c** and **d**, TMOKE spectra of MO SRR and equivalent material under s- and p-polarized incidence. (Solid lines: MO SRR, Dashed lines: equivalent material)

## Supplementary Note 9: Retrieved $\varepsilon$ and $\mu$ tensors of recent reported MO metamaterials

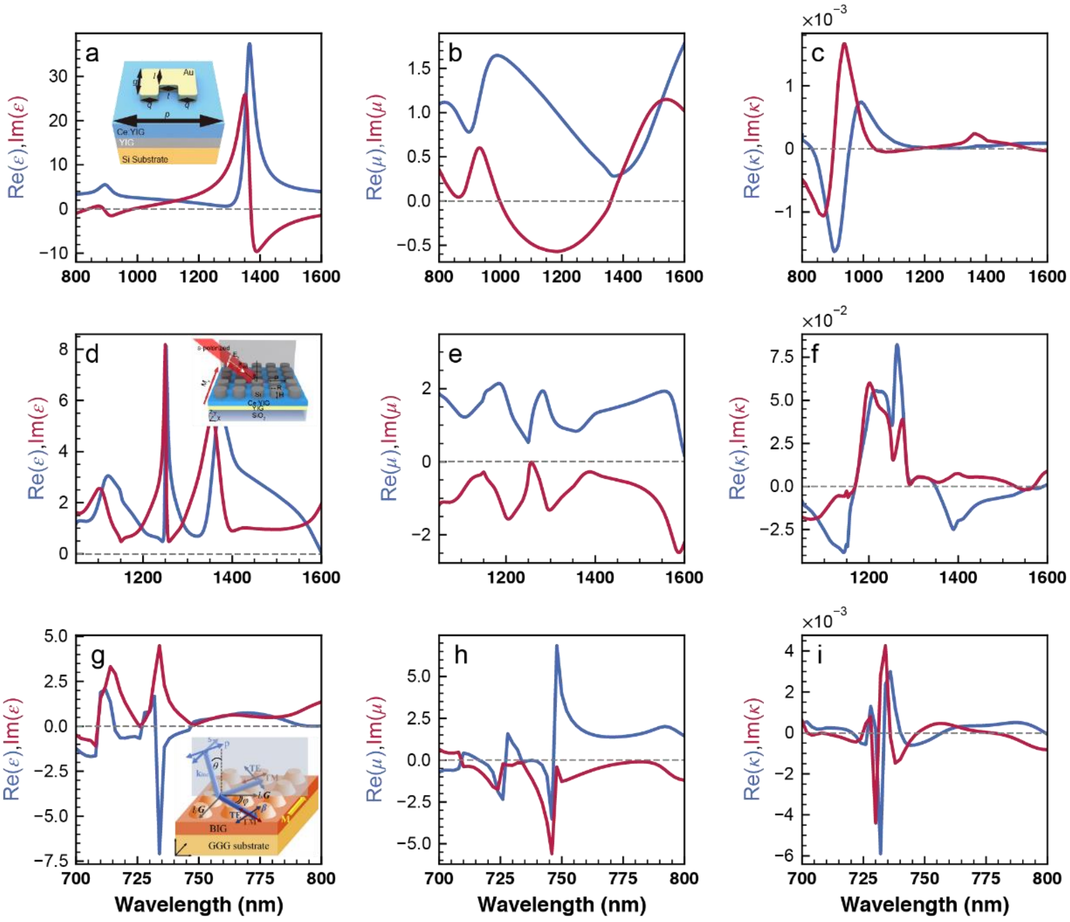


**Supplementary Figure 9. Retrieved $\varepsilon$ and $\mu$ tensors of three MO metamaterials.** Retrieved permittivity and permeability tensors spectra, **a**, **b**, **c**, of MO SRR (our work), **d**, **e**, **f**, of high index Mie resonators[13], **g**, **h**, **i**, of all-dielectric magnetic metasurface[14] (the results obtained in this paper are blue-shifted relative to the reported results, but the main result trends are consistent).

## Supplementary Note 10: Realization of gyroelectric properties at microwave frequencies

The same SRR structure can also be used to obtain p-polarized TMOKE in the microwave frequencies. As an example, we consider the magneto-optical metamaterial in Supplementary Figure 10a, where the copper SRR[10] are located on top of a gyromagnetic material $BaFe_{12}O_{19}$ (BaM). The microwave is incident with an angle in the *xoz* plane. The magnetic field $H_{ext}$ is applied along the *y* direction. The permeability of BaM can be expressed as:

$$\mu = \mu_0 \begin{bmatrix} \mu_r & 0 & -j\kappa \\ 0 & 1 & 0 \\ j\kappa & 0 & \mu_r \end{bmatrix} \tag{31}$$

Here, the $\mu_r$ is the diagonal elements and $\kappa$ is magneto-optical coefficients. The permeability tensor of BaM at microwave frequencies can be obtained from previous work[15] and the definition of the TMOKE is:

$$TMOKE = 2\frac{R_{P/S}(H+) - R_{P/S}(H-)}{R_{P/S}(H+) + R_{P/S}(H-)} \tag{32}$$

where $R_{P/S}(H\pm)$ is the reflectance of p-polarized (s-polarized) incidence under positive or negative applied magnetic field respectively. In planar BaM materials, the p-TMOKE would vanish because the magnetic field $\mathbf{H_y}$ of the electromagnetic wave is along $y$ direction, and the induced dynamic magnetization has nothing to do with $\kappa$. However, the SRR changes the local magnetic field distribution as shown by the arrows in Supplementary Figure 10a, causing non-zero $\mathbf{H_x}$ components, leading to a clear p-polarized TMOKE and gyroelectric properties. The simulated reflection and p-polarized TMOKE of the metamaterial is shown in Supplementary Figure 10b. The resonance at around 10 GHz corresponds to electric resonance, which is clearly accompanied with a non-zero p-polarized TMOKE.

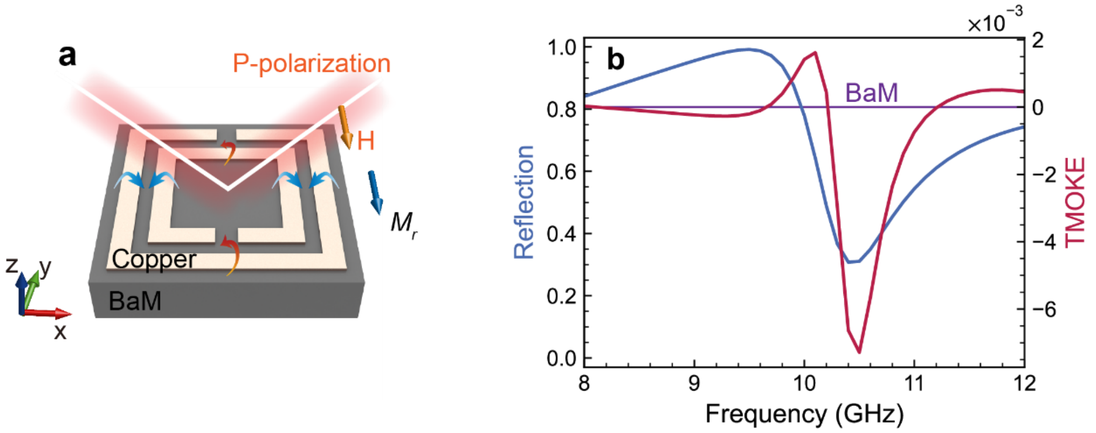


**Supplementary Figure 10. Schematic and function of the device. a**, Schematic of the metamaterial. The arrows show the magnetic field distributions. **b**, Reflection and TMOKE spectra of the MO-SRR on BaM under p-polarized incidence.

## Supplementary Reference


1. Zvezdin, A. K. & Kotov, V. a. c. A. *Modern magnetooptics and magnetooptical materials*. CRC Press (1997).
2. Dmitriev, V. Permeability tensor versus permittivity one in theory of nonreciprocal optical components. *Photon. Nanostr. Fundam. Appl.* **11**, 203-209 (2013).
3. Gurevich, A. G. & Melkov, G. A. *Magnetization oscillations and waves*. CRC press (2020).
4. Krinchik, G. & Chetkin, M. The problem of determining the dielectric permittivity and magnetic permeability tensors of a medium. *Sov. Phys. JETP* **36**, 1368-1369 (1959).
5. Pozar, D. M. *Microwave engineering*. John wiley & sons (2011).
6. Yeh, P. *Optical Waves in Layered Media*. Wiley (2005).
7. Menzel, C., Rockstuhl, C., Paul, T., Lederer, F. & Pertsch, T. Retrieving effective parameters for metamaterials at oblique incidence. *Phys. Rev. B* **77**, 195328 (2008).

8. Menzel, C., Paul, T., Rockstuhl, C., Pertsch, T., Tretyakov, S. & Lederer, F. Validity of effective material parameters for optical fishnet metamaterials. *Phys. Rev. B* **81**, 035320 (2010).
9. Koschny, T., Markoš, P., Smith, D. & Soukoulis, C. Resonant and antiresonant frequency dependence of the effective parameters of metamaterials. *Phys. Rev. E* **68**, 065602 (2003).
10. Depine, R. A. & Lakhtakia, A. Comment I on "Resonant and antiresonant frequency dependence of the effective parameters of metamaterials". *Phys. Rev. E* **70**, 048601 (2004).
11. Efros, A. L. Comment II on "Resonant and antiresonant frequency dependence of the effective parameters of metamaterials". *Phys. Rev. E* **70**, 048602 (2004).
12. Andryieuski, A., Malureanu, R. & Lavrinenko, A. Nested structures approach in designing an isotropic negative-index material for infrared. *J. Eur. Opt. Soc. Rapid Publ.* **4**, 09003 (2009).
13. Xia, S., *et al.* Circular displacement current induced anomalous magneto-optical effects in high index Mie resonators. Preprint at https://arxiv.org/abs/2108.00615 (2021).
14. Ignatyeva, D. O., *et al.* All-dielectric magnetic metasurface for advanced light control in dual polarizations combined with high-Q resonances. *Nat. Commun.* **11**, 5487 (2020).
15. Korolev, K. A., Wu, C., Yu, Z., Sun, K., Afsar, M. N. & Harris, V. G. Tunable ferromagnetic resonance in La-Co substituted barium hexaferrites at millimeter wave frequencies. *AIP Advances* **8**, 056440 (2018).
